# Research on brain functions related to visual information processing and body coordination function of pilots based on the low-frequency amplitude method

**DOI:** 10.3389/fnhum.2023.796526

**Published:** 2023-03-15

**Authors:** Kaijun Xu, Rui Liu, Xipeng Chen, Xi Chen, Yong Yang, Quanchuan Wang, Jiazhong Yang

**Affiliations:** School of Flight Technology, Civil Aviation Flight University of China, Guanghan, China

**Keywords:** pilots, resting-state fMRI, amplitude of low frequency fluctuation, frequency band, flight hours

## Abstract

**Objective:**

Research on the differences in physiological and psychological mechanisms of practitioners in different occupations is a current hot spot, such as pilots. This study explores the frequency-dependent changes of pilots’ low-frequency amplitudes in the classical frequency band and sub-frequency band between pilots and general occupations. The goal of the current work is to provide objective brain images for the selection and evaluation of outstanding pilots.

**Methods:**

Twenty-six pilots and 23 age-, sex-, and education-matched healthy controls were included in this study. Then the mean low-frequency amplitude (mALFF) of the classical frequency band and sub-frequency band was calculated. The two-sample *t*-test was performed on SPM12 to analyze the differences between the flight group and control group in the classic frequency band. To explore the main effects and the inter-band effects of the mean low-frequency amplitude (mALFF), the mixed design analysis of variance was applied in the sub-frequency bands.

**Results:**

Compared with the control group, left cuneiform lobe and the right cerebellum six area of pilots show significant difference in the classic frequency band. And the main effect results in the sub-frequency bands show that the area with higher mALFF in the flight group is located on the left middle occipital gyrus, the left cuneiform lobe, the right superior occipital gyrus, the right superior gyrus, and the left lateral central lobule. However, the area where the value of mALFF decreased is mainly located on the left rectangular cleft with surrounding cortex and the right dorsolateral superior frontal gyrus. Besides, compared with the slow-4 frequency band, the mALFF of the left middle orbital middle frontal gyrus of the slow-5 frequency band was increased, while the mALFF value of the left putamen, left fusiform gyrus, and right thalamus was decreased. The sensitivity of the slow-5 frequency band and the slow-4 frequency band to the pilots’ different brain areas was also different. Also, the different brain areas in the classic frequency band and the sub-frequency band were significantly correlated with pilots’ flight hours.

**Conclusion:**

Our findings showed that the left cuneiform brain area and the right cerebellum of pilots changed significantly during resting state. And there was a positive correlation between the mALFF value of those brain area and flight hours. The comparative analysis of sub-frequency bands found that the slow-5 band could elucidate a wider range of different brain regions, providing new ideas for exploring the brain mechanisms of pilots.

## 1. Introduction

Flight training has been identified as an essential task for flight safety, with the aim of high-quality development of civil aviation. Due to the rapid growth of total civil aviation transportation in China, safety risk pressure is increasing. Inconsistency between the behavioral response–based flight training mechanism of airlines and the profound changes in the operating environment, is becoming increasingly prominent. Research on the differences in physiological and psychological mechanisms of practitioners in different occupations is popular, especially for people who undertake extremely challenging tasks such as pilots. A full life cycle management system would be established for pilot skills (Civil Aviation Administration of China, 2019). The core competence of pilots needs to be comprehensively considered through theoretical analysis and training. The current flight training method involves subject-based training. After long-term flight training, pilots have corresponding changes in their brain function and structure, which could lead to changes in personal abilities. Pilot is the direct controller of an aircraft, and the flight expertise to handle an emergency situation has a significant impact on the operation of an aircraft. After the extensive application of computer technology to the aviation industry, the integration of on-board electronic equipment has made the operation of aircrafts automated and intelligent. To a certain extent the safety and reliability of aircrafts can be greatly improved (Denney and Pai, 2014; Lališ et al., 2018). Although the safety and reliability of aircrafts have matured over time, the occurrence of aviation accidents is still unavoidable. According to statistics, the overall rate of aircraft accidents induced by human factors are declining, but flight accidents induced by human factors still exist, and the proportion of relative mechanical failures is gradually increasing (Casner and Jonathan, 2014; Kharoufah et al., 2018). More than 60% of flight accidents are directly or indirectly caused by human factors (Boyd, 2017; Erjavac et al., 2018). Therefore, it is of great practical significance to study the physiological and psychological mechanisms of pilots.

Most of the researches on pilots focuses on the psychological qualities of pilots (Hunter et al., 2011; You et al., 2013; Tsifetakis and Kontogiannis, 2019), while there are few studies aiming at the advanced neural mechanisms of pilots (Adamson et al., 2012, 2014; Causse et al., 2013; Ahamed et al., 2014; Toppi et al., 2016; Chen X. et al., 2019). Previous study found that the default mode network (DMN) in pilots’ brains has changed, and the connectivity of the DMN is enhanced through pilots’ flight training (Chen X. et al., 2019). Adamson et al. (2014), Taylor JL and others studied the brain function of pilots and found that the activation of the bilateral caudate nucleus of skilled pilots was reduced during landing tasks. Aviat Space Environ Med et al. found that pilots’ hippocampi tend to grow with their flight experience increasing, and the size of the hippocampi can change in response to intensive training (Adamson et al., 2012). Liu et al. (2015), discussed the application of resting-state functional magnetic resonance imaging (rs-fMRI), fractional amplitude of low frequency fluctuation (fALFF) and regional homogeneity (ReHo) methods in the assessment of resting brain function in pilots after hypoxia exposure.

Resting-state functional magnetic resonance imaging technology allow researchers to visualize the local brain functional and is widely used in brain science research. The amplitude of low-frequency fluctuation is one of the widely recognized fMRI research methods, which reflects the strength of neuron activity by obtaining the average value of the amplitude at a frequency point in a specific frequency band (Zang et al., 2007). Researchers have divided the low-frequency amplitude into five sub-frequency bands according to the frequency range (Zuo et al., 2010): slow-6 (0∼0.01 Hz), slow-5 (0.01∼0.027 Hz), slow-4 (0.027∼0.073 Hz), slow- 3 (0.073∼0.198 Hz), and slow-2 (0.198∼0.25 Hz). Among these sub-frequency bands the slow-6, slow-3, and slow-2 frequency bands, respectively represent frequency drift, white matter signal and physiological noise, while the slow-5 frequency band and the slow-4 frequency band represents the gray matter signal of the brain. The mALFF analysis method is used in the slow-5 frequency band and the slow-4 frequency band to explore the sensitivity of pilots’ neuronal activity in a specific frequency band and changes in spontaneous activity in pilots’ brain.

## 2. Materials and methods

### 2.1. Participants

The subjects in the flight group were selected from the Civil Aviation Flight University of China. A total of 26 instructors and in-service pilots of various airlines, all of whom were male, aged 22∼36(26.1 ± 3.3), had 214∼9800(1225.7 ± 2343.8) cumulative flying hours, and 16 years of education. In addition, 23 healthy subjects participated in the work were selected as the control group, all of whom were male, aged 23∼37(29.9 ± 4.0) with 16 years of education.

Inclusion criteria: All subjects had a bachelor’s degree, right-handed, and had no head trauma. Also, neurological diseases or medical history do not exist in themselves and their first-degree relatives. The experimental procedure (no. 2018-042002) was approved by the Ethics Committee of the University of Electronic Science and Technology of China, and all subjects had signed an informed consent form.

### 2.2. Imaging data acquisition

The MRI scan equipment for all subjects in this experiment was an 8-channel GE 3.0T MR750 MRI magnetic resonance instrument at the Magnetic Resonance Imaging Centre of the University of Electronic Science and Technology of China. The subject lay supine, rested with his eyes closed to avoid thinking about specific problems. The subject’s head was fixed with foam pads and other objects to reduce or prevent head movement. The head axial scan used the canthal line as the scanning baseline, and all lights in the MRI room were turned off to prevent light from affecting the subject. A standard gradient echo pulse sequence was used to acquire functional magnetic resonance images. The scanning parameters are as follows: repetition time 2,000 ms, echo time 30 ms, flip angle 90°, scanning matrix 64 × 64, scanning field of view 24cm × 24cm, and slice thickness 4 mm (layer interval 0). A total of 255 whole brain scans were collected for each subject, and 35 layers of the whole brain were scanned each time.

### 2.3. Resting state fMRI data pre-processing

The format of the data scanned by the nuclear magnetic resonance instrument is DICOM. dcm2nii software was used to convert the original image data for subsequent calculations. The first 10 time points of each subject’s data were removed to eliminate the influence of the initial scan due to the uneven magnetic field. Based on the MATLAB 2013b platform, the SPM12 (Statistical Parametric Mapping 12)^1^ toolbox was used to pre-process the remaining time point data with the following steps: ➀ time layer correction; ➁ head movement correction, according to the head movement correction curve, by eliminating head movement translation >2.0 mm and (or) rotation >2.0° data (1 person in the pilot group and 0 person in the control group); ➂ spatial standardization, with individual brain image data referencing the spatial coordinate system of the Montreal Neurological Institute (MNI) standard human brain template, standardized space 3mm × 3mm × 3mm; ➃ spatial smoothing was performed with a Gaussian kernel with full width at half maximum (FWHM); and ➄ linear drift was removed.

### 2.4. Mean low-frequency amplitude calculation

Amplitude of low frequency fluctuation calculation was performed based on the pre-processed data. The power spectrum was obtained by transforming the time series of each voxel into the frequency domain through a Fourier transform, and the ALFF value was obtained by the square root of the pre-set frequency domain. To eliminate the difference in the overall level of ALFF between individuals, the ALFF value of each voxel was divided by the mean ALFF value of the whole brain, and each voxel in the whole brain was standardized in turn to obtain the mean ALFF, namely, mALFF. The mALFF values of the flight group and the control group in the slow-5 frequency band (0.01∼0.027 Hz) and slow-4 frequency band (0.027∼0.073 Hz) were calculated for statistical analysis.

### 2.5. Statistical analysis

The SPM12 software package based on MATLAB2013b was used to perform mixed design analysis of variance (ANOVA) on each group of data. Differences between groups were limited to the results of the one sample *t*-test (FWE correction), and Gaussian random-field (GRF) multiple comparison correction was used to correct the statistical results. The main effects between the flight group and the control group, as well as the main effects between the slow-5 and slow-4 frequency bands, were compared. The results were corrected by GRF (voxel level *P* < 0.001), and the results were superimposed on the Colin27 template. The results are displayed. With SPM12, two-sample *t*-test was performed on the data of the flight group and the control group in the classical frequency band and slow-5 and slow-4 frequency bands, and the results were corrected by GRF (voxel level *P* < 0.01).

## 3. Results

The demographic data comparison between the two groups is shown in Table 1.

**TABLE 1:** Comparison of demographic data between the two groups ($\bar{x}\pm \text{s}$).

Characteristics	Pilots (*N* = 26)	Controls (*N* = 24)	*T*-value	*P*-value (two-tailed)
Age (years)	26.1 ± 3.3	29.9 ± 4.0	−3.5	0.001
Sex (% male)	100	100	–	–
Education (years)	16	16	–	–
Handedness (% right)	100	100	–	–
Total flight time (hours)	1225.7 ± 2343.8	–	–	–

### 3.1. Results of main effect analysis between groups

**FIGURE 1:**
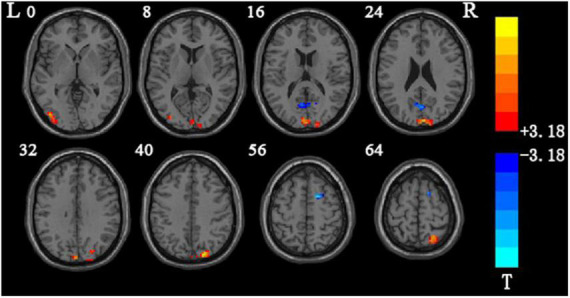
Brain areas showing the main effect analysis results between groups.

Compared with the values in the control group, the results of the main effect analysis between groups showed that the mALFF values of the left middle occipital gyrus, left cuneiform lobe, right superior occipital gyrus, right superior gyrus, and left side central lobule are increased in the flight group. The mALFF value of the rectangular fissure and the surrounding cortex and the right dorsolateral superior frontal gyrus decreased (Table 2 and Figure 1).

**TABLE 2:** Main effect analysis results between groups.

Brain regions (AAL)	Cluster size (Voxel number)	Centre (MNI)			*t*-value	*p*-value
		**x**	**y**	**z**		
Occipital_Mid_L	49	−42	−81	0	4.6785	0.001
Cuneus_L	169	0	−93	21	5.0384	0.001
Calcarine_L	80	3	−69	21	−4.5959	0.001
Occipital_Sup_R	58	21	−84	39	5.3521	0.001
Frontal_Sup_R	40	18	6	60	−5.1106	0.001
Parietal_Sup_R	40	24	−63	63	4.5966	0.001
Paracentral_Lobule_L	58	−12	−3	78	4.5903	0.001

### 3.2. Results of main effect analysis between frequency bands

**FIGURE 2:**
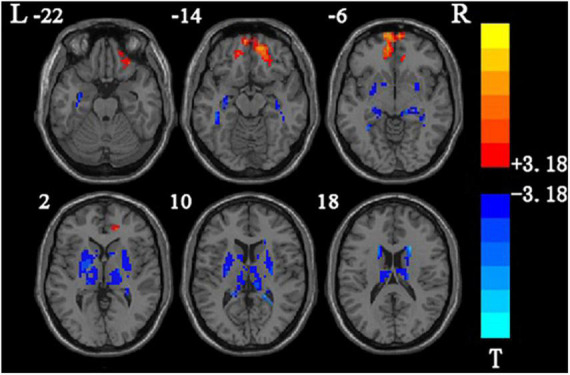
Brain areas showing the main effects between frequency bands.

The analysis of the main effects between frequency bands showed that the specificity of the main effects between frequency bands are in the left middle orbital frontal gyrus, left fusiform gyrus, left putamen, and right thalamus (Table 3 and Figure 2).

**TABLE 3:** Main effect analysis results between frequency bands.

Brain regions (AAL)	Cluster size (Voxel number)	Centre (MNI)			*t*-value	*P*-value
		**x**	**y**	**z**		
Frontal_Mid_Orb_L	327	−6	60	−12	5.0006	0.001
Fusiform_L	48	−33	−48	−6	−5.1907	0.001
Putamen_L	547	−21	−24	24	−8.3436	0.001
Thalamus_R	546	21	3	24	−7.2588	0.001

### 3.3. Sub-band analysis results

After the two-sample *t*-test analysis between groups, compared with the control group, the brain areas with increased mALFF values in the classic frequency flight group were the left cuneiform lobe and the right cerebellum 6. No brain areas with reduced mALFF values were found (Table 4). The brain areas where the mALFF value increased in the slow-4 band in flight group were the left cuneiform lobe, and no brain area where the mALFF value decreased was found (Table 5).

**TABLE 4:** Classic frequency band (0.01∼0.08 Hz) results between groups of brain areas.

Brain regions (AAL)	Cluster size (Voxel number)	Centre (MNI)			*t*-value	*P*-value
		**x**	**y**	**z**		
Cuneus_L	170	21	−84	39	3.9030	0.01
Cerebelum_6_R	111	15	−75	−24	4.3722	0.01

**TABLE 5:** Slow-4 frequency band (0.027∼0.073 Hz) results between groups of brain areas.

Brain regions (AAL)	Cluster size (Voxel number)	Centre (MNI)			*t*-value	*P*-value
		**x**	**y**	**z**		
Cuneus_L	166	21	−84	39	3.8256	0.01

The brain areas with significantly higher mALFF values in the slow-5 band of the flight group included the right cerebellar area 6, the left cuneiform lobe, and the left lateral central lobule. The mALFF value of the left sphenoid fissure and its surrounding cortex decreased (Table 6). The mALFF values of brain regions with differences between the flight groups in the classic frequency band, the slow-4 frequency band and the slow-5 frequency band were extracted, and the Pearson correlation calculation was carried out with the flight time of the flight group.

**TABLE 6:** Slow-5 frequency band (0.01∼0.027 Hz) results between groups of brain areas.

Brain regions (AAL)	Cluster size (Voxel number)	Centre (MNI)			*t*-value	*P*-value
		**x**	**y**	**z**		
Cerebelum_6_R	159	21	−75	−24	4.5122	0.01
Cuneus_L	113	3	−90	30	4.4098	0.01
Paracentral_Lobule_L	151	−3	−36	63	3.6619	0.01
Calcarine_L	155	−6	−60	3	−4.1805	0.01

**FIGURE 3:**
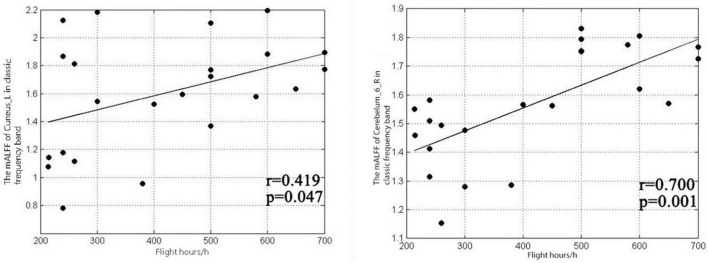
Correlation between mALFF values and flight duration in the left cuneiform lobe and right cerebellum 6 area in the classic frequency band.

**FIGURE 4:**
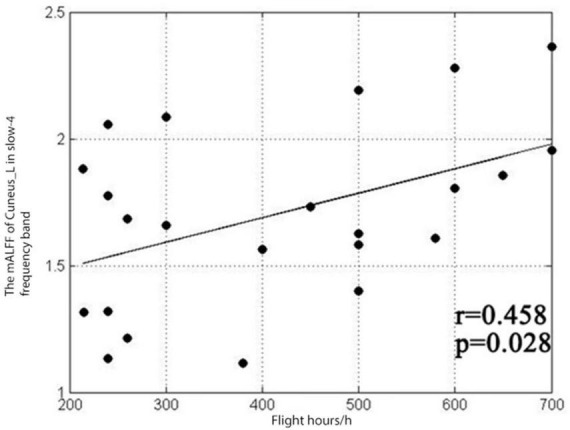
Correlation between the mALFF value at the left wedge of the slow-4 frequency band and the flight duration.

**FIGURE 5:**
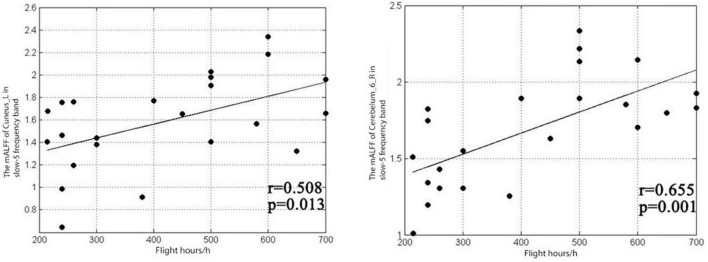
Correlation between mALFF values and flight duration in the left cuneiform lobe and the right cerebellum 6 area in the slow-5 frequency band.

The results showed that the mALFF value of the left cuneiform of pilots in the classic frequency band was positively correlated with the number of flight hours (*r* = 0.419, *P* = 0.047), and the mALFF value of the right cerebellar area 6 was positively correlated with the number of flight hours (*r* = 0.7, *p* = 0.001), as shown in Figure 3. The pilot’s left cuneiform mALFF value in the slow-4 frequency band was positively correlated with its flight hours (*r* = 0.458, *P* = 0.028), as shown in Figure 4. Regarding the pilot’s left cuneiform of mALFF value and its flight time in the slow-5 frequency band, the duration was positively correlated (*r* = 0.508, *P* = 0.013), and the mALFF value of the right cerebellum 6 area was positively correlated with the flight time (*r* = 0.655, *P* = 0.001), as shown in Figure 5.

## 4. Discussion

### 4.1. Comparison between the low-frequency oscillation in FMRI and the neural oscillation in EEG

Many authoritative researchers at home and abroad have found that different brain regions have varying sensitivity to different frequency band oscillation signals (Han et al., 2021a,b,c, 2022a,b,c; Cao et al., 2022). EEG is often used to study neuronal activity. EEG signals are obtained by recording electrical signals from cortical pyramidal neurons. So, EEG signals from subcortical area are easily missed. The amplitude of EEG is a reflection of the continuous change of neural electrical activity in a short time, and is the amplitude of electrical signals in the cortex. However, due to the influence of cerebrospinal fluid in the conduction process, the activity is more complex. Not only is EEG generally insensitive to deep brain regions, but the waveform also varies with brain location. EEG is typically characterized by frequency bands of <4 Hz (deltaδ), 4–7 Hz (thetaθ), 7–13 Hz (alphaα), 13–30 Hz (betaβ), and >30 Hz (gammaγ) (Chen W., 2021). The Delta rhythm is a prominent feature of deep sleep but is also observed during high cognitive demands and some brain lesions; Theta rhythms can be observed during sleepiness and are often present in the sleep-deprived brain; The Alpha rhythm reflects the relaxed wakefulness with eyes closed; Beta and gamma rhythms are most pronounced during cognitive tasks (Snipes et al., 2022). fMRI utilizes a gradient magnetic field to encode the space, which can directly measure the blood oxygen metabolism of the brain, so the effects of cerebrospinal fluid, skull and scalp could be not considered. The functional connectivity of fMRI is highly correlated with the synchronization of spontaneous oscillations of neural activity, which can reflect the connectivity between multiple brain regions, and there is symmetry between the two hemispheres (Li, 2020; Wang, 2021). fMRI is an indirect observation of neural activity by measuring metabolic and hemodynamic response signals, accompanied by neurovascular coupling and physical measurement of MRI. Thus, the amplitude of fMRI is the amplitude of the magnetic signal. mALFF analysis, as one of the commonly used research methods of resting-state functional magnetic resonance imaging, can reflect the spontaneous activity of local neurons in the brain. Most studies on ALFF mainly focus on low-frequency oscillation signals in the frequency band of 0.01–0.08 Hz, while mALFF sub-frequency band analysis is mostly used in disease research (Hoptman et al., 2010; Li et al., 2016; Zhang et al., 2016), and the research on the pilot’s resting state is relatively rare. Therefore, the research on the pilot’s mALFF in different frequency bands in the resting state is an exploratory study. In our study, mALFF analysis was used to explore the changes of pilots’ brain mALFF in resting state, and it was found that the neuronal activity in different brain regions was frequency-dependent.

### 4.2. Analysis of the main effect differences between groups

Through the analysis of the main effects between groups, it was found that the mALFF value of the left middle occipital gyrus, left cuneiform lobe, right superior occipital gyrus, right superior gyrus, the left square cleft and left lateral central lobule increased in the flight group. The mALFF value of the peripheral cortex and the right dorsolateral superior frontal gyrus were decreased. As a result, the brain areas of interest were mainly concentrated in the DMN. With flight experience increasing, pilots’ brain function has changed, which led to a change in the pilots’ DMN. The DMN is mainly composed of the posterior cingulate cortex (PCC)/praecuneus, medial prefrontal cortex (MPFC), inferior parietal lobule (IPL), and bilateral temporal cortex. PCC is considered to be the core node of the DMN and participates in the awakening function of maintaining consciousness (Leech et al., 2012). The PCC is also related to the adjustment of concentration and attention span (Leech and Sharp, 2014). When operating an aircraft, any small mistake or decision-making error during take-off and landing has serious consequences. Therefore, it is necessary for a pilot to maintain a high degree of vigilance, which causes the pilot’s PCC to exhibit higher activity level than ordinary ground personnel. The parietal lobe plays an important role in visual motor control, attention and eye movement control (Wang, 2019); the rostral gyrus involves sensory, visual and spatial perception functions (Chen Y., 2021; Yang, 2021). Pilots have a more developed optic nerve than ordinary people through long-term flight training. At the same time, this study found that pilots have a better perception of time, frequency and moving objects than ordinary people (Zhang, 2016). In this study, it was found that different effects between groups were located in the DMN, revealing that pilots have a higher degree of spontaneous activity in the DMN in the resting state, which is also consistent with the conclusions of previous studies (Chen X. et al., 2019).

### 4.3. Analysis of the results of the main effect difference between frequencies

The analysis of the main effects between the slow-5 frequency band and the slow-4 frequency band showed that compared with the slow-4 frequency band, the area with higher mALFF value in the slow-5 frequency band was mainly located in the bilateral orbital middle frontal gyrus. Compared with the frequency band, the brain areas with higher mALFF values in the slow-4 frequency band were the left fusiform gyrus, bilateral putamen, bilateral thalamus, and bilateral hippocampus. Xue found that compared with the slow-5 frequency band, the slow-4 frequency band increased the mALFF value in the bilateral thalamus, left caudate nucleus, and left central posterior gyrus and decreased in the posterior cingulate gyrus region. Part of the results are consistent (Xue et al., 2014). According to previous studies, low-frequency oscillations achieved the integration of larger neuron networks, while high-frequency frequencies have lower energy and can only be confined to smaller neural spaces (Buzsáki and Draguhn, 2004). Although the physiological functions and mechanisms of neuronal activity in the slow-5 and slow-4 frequency bands have not been fully elucidated, there is a competitive relationship between adjacent frequencies (Han et al., 2011; Zhu et al., 2015; La et al., 2016). This relationship also proves the frequency specificity of neuronal activity in different brain regions (Zou, 2019). This study found that most of the areas with higher mALFF values in the slow-4 band are in the subcortex (bilateral thalamus, bilateral hippocampus), and it is speculated that the slow-4 band is more sensitive to detecting brain activity in the subcortical brain areas.

### 4.4. Amplitude of low frequency fluctuation frequency band related changes

Through statistical analysis of the two-sample *t*-test, the results of the classic frequency band mALFF value between groups show that in the 0.01∼0.08 Hz classic frequency band, the areas where the mALFF value of the flight training group has significantly increased compared with the control group are the left and right cuneiform. Area six of the lateral cerebellum indicate that the cuneiform lobe and the right cerebellar area are more active in the resting state in pilots than in ordinary people. Anatomically speaking, the cuneiform lobe is located between the rectangular cleft and the parieto-occipital fissure. It belongs to the occipital lobe in the “three grooves and five lobes” of the brain. It is the primary visual cortex of the brain and is also an important part of the visual network, involved in visual spatial information processing and execution. The cuneiform lobes can receive the information transmitted from the retina and process the visual information transmitted by the retina in the visual transmission pathway which play an important role in information processing and execution (Zheng, 2021). In the early stage, SchraaTam and others found that the cuneiform lobe of the brain also has an important function in the eye movement reflex, which can stabilize the image on the retina. If the cuneiform lobe function of the brain changed, it will cause eye movement disorders (Schraa-Tam et al., 2009). Pilots are required to monitor the instruments in real time during the take-off, cruise, and landing phases. In different stages of flight, the areas where the pilots’ eye-concentrated moving are also different. Especially in the event of a single engine failure, pilots’ gaze rate greatly increased, the time of gazing at the instrument was reduced, and the scan length was increased (Chen B. et al., 2019). To deal with the search for emergency information after single engine failure, according to the rapid flat sweep data collected, the instrument state generates emergency strategies to ensure the smooth landing of the aircraft. Through the monitoring training of instruments and meters in daily flight and the single-engine failure emergency training in the simulator, the brain areas related to eye movement and visual processing were shown to be enhanced. Therefore, it is speculated that the neurons in the cuneiform lobe on the left side of the flight group are spontaneously activated. The reason the degree in the pilot group is higher than that of the control group is also related to the daily flight training of the pilots. At the same time, pilots also have higher spontaneous activation performance in area 6 of the right cerebellum. Anatomically speaking, the cerebellum in the posterior fossa is composed of cerebellar vermis, the cerebellar hemisphere and cerebellar tonsil. As a large “regulator,” the cerebellum participates in the adjustment of body balance, muscle tension and voluntary movement. At the same time, its function also includes fine adjustment of the eyes (Fu, 2007). Studies have shown that the posterior lobe of the cerebellum plays a key role in movement control and perception, especially in the control of eye movements (Buckner, 2013), and studies have shown that the cerebellum has a regulatory effect on the execution of fine eye movements (Striemer et al., 2015). The mALFF values in area six of the cerebellum on the right side of the pilot was higher than that of ordinary professionals, indicating that the spontaneous activation of the cerebellum was higher. Similar to cuneiform function, both the cerebellum and cuneiform are involved in the adjustment of eye movements, and the enhancement of their spontaneous activity intensity is also related to daily flight training.

According to the results of sub-frequency band research, the left cuneiform lobe shows higher activation in the slow-5 and slow-4 bands. My previous research found that the left cuneiform lobe is more activated in the 0.01∼0.08 Hz classic frequency band. Gao’s conclusion is consistent (Xu et al., 2020). However, the right cerebellum, the left paracentral lobule, the left trellis fissure and the surrounding cortex showed significant differences only in the slow-5 frequency band. The slow-5 frequency band and the slow-4 frequency band are sensitive to the spontaneous activities of different brain regions. The slow-5 frequency band can reveal a wider range of different brain areas than other frequency bands, and it can be used as a sensitive indicator to explore the special brain mechanisms of pilots.

At the same time, it was found that pilots’ mALFF value of the left cuneiform lobe in the slow-4 frequency band was positively correlated with the flight duration (*r* = 0.458, *P* = 0.028). In the slow-5 band, pilots’ left cuneiform mALFF value was positively correlated with flight duration (*r* = 0.508, *P* = 0.013). The mALFF value of right cerebellar area six was positively correlated with flight duration (*r* = 0.655, *P* = 0.001).

Through long-term flight training, pilots tend to strengthen their ability to monitor instrument information during flight training, which in turn improves the efficiency of their eye movement and their concentration. Especially in the take-off and landing phases of aircraft, the capture of moving objects and the perception of acceleration enhances the pilot’s brain function and visual processing. In daily roller and spiral ladder training, physical coordination and body balance are further enhanced, and the related brain functions are also enhanced. Corresponding conclusions are also obtained through the correlation analysis of experimental data and flight duration.

## 5. Conclusion

In summary, in the resting state of pilots, the spontaneous activities of the brain areas related to visual information processing and coordinated movement of the body are significantly enhanced. Also, pilots’ DMN brain function has changed significantly compared with that of ordinary professionals, which reflects the importance of the DMN in the neurophysiological mechanism of pilots. The intensity of spontaneous activity in some areas of pilots’ brains is also frequency-dependent, showing higher sensitivity in the slow-5 frequency band. This study provides ideas for additional studies of pilot brain network mechanisms.

## Data availability statement

The datasets presented in this study can be found in online repositories. The names of the repository/repositories and accession number(s) can be found below: https://pan.baidu.com/s/12VhqdqZ5z7QYtYqVUbHmlg?pwd=ptnn.

## Ethics statement

The studies involving human participants were reviewed and approved by the Ethics Committee of the University of Electronic Science and Technology of China (Chengdu, China). The patients/participants provided their written informed consent to participate in this study.

## Author contributions

XC, KX, and QW contributed to conception and design of the study. XC and YY organized the database. XPC, XC, and RL performed the statistical analysis. RL wrote the first draft of the manuscript. KX and XPC wrote sections of the manuscript. All authors contributed to manuscript revision, read, and approved the submitted version.

## Footnotes

1
http://www.fil.ion.ucl.ac.uk/spm/
